# Whipworm-Associated Intestinal Microbiome Members Consistent Across Both Human and Mouse Hosts

**DOI:** 10.3389/fcimb.2021.637570

**Published:** 2021-03-11

**Authors:** Bruce A. Rosa, Caroline Snowden, John Martin, Kerstin Fischer, Jonah Kupritz, Ethiopia Beshah, Taniawati Supali, Lincoln Gankpala, Peter U. Fischer, Joseph F. Urban, Makedonka Mitreva

**Affiliations:** ^1^ Division of Infectious Diseases, Department of Medicine, Washington University School of Medicine, St. Louis, MO, United States; ^2^ U.S. Department of Agriculture, Agricultural Research Service, Beltsville Agricultural Research Center, Animal Parasitic Diseases Laboratory, Beltsville, MD, United States; ^3^ Department of Parasitology, Faculty of Medicine, Universitas Indonesia, Jakarta, Indonesia; ^4^ Public Health and Medical Research, National Public Health Institute of Liberia, Charlesville, Liberia; ^5^ Department of Genetics, Washington University School of Medicine, St. Louis, MO, United States; ^6^ McDonnell Genome Institute, Washington University in St. Louis, St. Louis, MO, United States

**Keywords:** microbiome, intestinal microbiota, helminth, whipworm, animal model

## Abstract

The human whipworm *Trichuris trichiura* infects 289 million people worldwide, resulting in substantial morbidity. Whipworm infections are difficult to treat due to low cure rates and high reinfection rates. Interactions between whipworm and its host’s intestinal microbiome present a potential novel target for infection control or prevention but are very complicated and are identified using inconsistent methodology and sample types across the literature, limiting their potential usefulness. Here, we used a combined 16S rRNA gene OTU analysis approach (QIIME2) for samples from humans and mice infected with whipworm (*T. trichiura* and *T. muris*, respectively) to identify for the first time, bacterial taxa that were consistently associated with whipworm infection spanning host species and infection status using four independent comparisons (baseline infected vs uninfected and before vs after deworming for both humans and mice). Using these four comparisons, we identified significant positive associations for seven taxa including *Escherichia*, which has been identified to induce whipworm egg hatching, and *Bacteroides*, which has previously been identified as a major component of the whipworm internal microbiome. We additionally identified significant negative associations for five taxa including four members of the order *Clostridiales*, two from the family *Lachnospiraceae*, including *Blautia* which was previously identified as positively associated with whipworm in independent human and mouse studies. Using this approach, bacterial taxa of interest for future association and mechanistic studies were identified, and several were validated by RT-qPCR. We demonstrate the applicability of a mouse animal model for comparison to human whipworm infections with respect to whipworm-induced intestinal microbiome disruption and subsequent restoration following deworming. Overall, the novel cross-species analysis approach utilized here provides a valuable research tool for studies of the interaction between whipworm infection and the host intestinal microbiome.

## Introduction

The intestinal microbiota has a significant impact on human physiology, and it has been demonstrated to modulate immune function, growth (Subramanian et al., 2014; Lim et al., 2015; Subramanian et al., 2015), metabolism, and overall health (Guinane and Cotter, 2013). Soil transmitted helminths (STH) residing in the host intestine can directly affect the immune system (Else, 2005; Altmann, 2009; Elliott and Weinstock, 2012) and may indirectly influence it by affecting the intestinal microbiota and the mucosa. Children are often exposed to infections (especially large roundworm and whipworm) prior to the stabilization of their microbiota (at around 3 years of age (Yatsunenko et al., 2012)), resulting in disruption of normal microbial community development and long-term dysbiosis and malnourishment due to reduced absorption of nutrients (Glendinning et al., 2014). Therefore, as a modifiable part of human “pan-genome”, it may be possible to prudently manipulate an at-risk host microbiome through simple and inexpensive nutritional strategies (e.g. *via* inclusion of local fermented food) that supplement therapeutic use of anthelmintics. However, the role of commensal bacteria in STH infections is not well understood, although it is recognized that active cross-kingdom talk occurs.

Among the three most prevalent STHs (hookworm, large roundworm and whipworm), whipworm (*Trichuris trichiura*) infects over 289 million people mainly in Sub-Saharan Africa, India, China, East Asia, and South America, resulting in an estimated 212,700 years living with disability (YLD) (GBD, 2018). Whipworm eggs hatch to L1 stage in the large intestine (caecum and/or proximal colon), after which they penetrate the epithelium and form multicellular epithelial ‘tunnels’, which are poorly understood (Else et al., 2020). From there, they molt to adult stage, but extend their posterior end into the lumen while keeping the anterior end embedded in the epithelial layer (Else et al., 2020). Whipworm infections are correlated with impairments in growth and cognitive development in children (preschool and school-age), impairments in the health and well-being of women and increases in the risk of adverse maternal and neonatal outcomes (Nokes and Bundy, 1994). To reduce morbidity due to infections (Brooker et al., 2015), the US Agency for International Development and the World Health Organization (WHO) support mass drug administration (MDA) to treat at least 75% of all at-risk children by the year 2020 and to reduce maternal anemia by 50% by 2025 (World Health Organization, 2014). However, it is not expected that these measures will lead to sustainable control or elimination of whipworm infection, since prevalence remains high due to very high rates of reinfection post-treatment [82% (Jia et al., 2012)] and low and variable cure rates [~50% (World Health Organization, 2008)] especially after treatment with a single anthelmintic drug. Furthermore, anthelmintic drug resistance is a potential future risk for STH control programs, since benzimidazole resistance is already widespread in veterinary helminths (specifically against albendazole and mebendazole, which represent the primary drugs used for MDAs) (Kaplan, 2004). There is an urgent need to develop sustainable and integrated helminth control strategies as alternatives (or complementary) to MDA, especially in developing economies unable to support the necessary infrastructure and sanitation interventions.

There are a limited number of published papers studying human microbiome members associated with whipworm infections and these papers report disparate results. One study from Ecuador failed to identify significant associations between STHs (*T. trichiura* and *Ascaris lumbricoides*) and the fecal microbiome (Cooper et al., 2013); a study on Malaysian subjects identified several bacterial taxa associated with inflammation (Ramanan et al., 2016), and another study from Malaysia identified microbiome taxa (and blood markers) associated with whipworm infections (Lee et al., 2019). A study from Sri Lanka identified several taxa significantly associated with hookworm, whipworm, or roundworm infection (Jenkins et al., 2017). Animal models have also proven useful for studying whipworm infections, and previous studies have utilized mice models to study microbiome interactions with the mouse whipworm (*T. muris*) (Holm et al., 2015; Houlden et al., 2015; White et al., 2018; Schachter et al., 2020).

Unfortunately, translational research on the role of commensal bacteria in whipworm infections has been impeded by knowledge gaps in cross-kingdom molecular interactions across host species. For example, antibiotic treatment protects against the establishment of *T. muris* in mice, and bacteria are thought to provide molecules necessary for hatching of *T. muris* ova (Hayes et al., 2010). Conversely, bacteria and their products can enhance the mucosal barrier to protect from invading parasites, and support host repair mechanisms to reduce pathology, which collectively suggests the existence of active parasite-host-microbial interactions (Hayes et al., 2010; Dawson et al., 2020). In pigs, *T. suis* infection affects the abundance of ~13% of bacteria in the proximal colon in a worm burden-dependent manner (Wu et al., 2012). In a previous study, we analyzed microbiome assemblages during moderate and heavy STH infections in Indonesia and Liberia and for the first time identified specific members of the intestinal microbiome that discriminate between STH-infected and non-infected states across very diverse geographical regions using multiple statistical methods (Rosa et al., 2018). We also detected microbiome-encoded biological functions potentially associated with STH survival strategies. However, there has been little research to date that integrates datasets across species.

While previous results provide insight into cross-kingdom interactions, key mechanistic studies in both humans and model organisms are necessary to produce translational discoveries. We therefore urgently need a comprehensive understanding of common bacterial taxa that modulate or are altered by STH infection in humans as well as in relevant animal models. Here, we compare the microbiome of samples from humans with exclusive whipworm infections (Rosa et al., 2018) to newly generated samples from mice infected with whipworm. We use a 16S rRNA gene analysis pipeline to both datasets simultaneously to identify and compare bacterial taxa members consistently associated with whipworm infection (at baseline and following deworming) spanning human and mouse host species. A subset of taxa was validated by real-time quantitative PCR (RT-qPCR). This not only highlights the importance of these specific bacterial taxa but also highlights the usefulness of the mouse whipworm infection model for future mechanistic studies, holding potential for translational applications in humans.

## Materials and Methods

### Sample Sources

For the mouse experiments, mixed sex C57BL/6N/STAT6KO mice were inoculated per os with 250 infective *T. muris* eggs. Mice were derived from breeding pairs of B6.129S2(c)-Stat6(tm1Gru)/J purchased from Jackson Laboratories. After 35 days, stool samples were collected from 18 infected and 18 uninfected mice (“baseline” infection samples). Six of these infected mice were treated per os with a combination of mebendazole (Fluka, City, Country) used at 80 mg/kg body weight (solution prepared at 50 mg/ml in 10% Tween 20 + dH_2_O) and ivermectin (used at 4 mg/kg body weight; Vetrimec 1%; MWI Animal Health, City, Country) to deworm them. Fecal samples were collected from these mice after an additional 19 days, on day 54 (“dewormed” samples). Control mice which were (i) uninfected and received no anthelmintic treatment, (ii) uninfected and received anthelmintic treatment, and (iii) infected and received no anthelmintic treatment (N=6 each) were also included in the dataset.

Human stool samples from Indonesia were retrieved for analysis from our previous study (Rosa et al., 2018) and collected as previously described (Wiria et al., 2010) (sequenced on the Genome Sequencer Titanium FLX [Roche Diagnostics, Indianapolis, Indiana] to an average of 6,000 reads per sample). Samples were collected in Flores island Indonesia between 2008 and 2010, and patients received albendazole treatment every 3 months from 2008 to 2010 (Rosa et al., 2018). Only the subset of these samples that were uninfected or exclusively *T. trichiura* infected were used for the “baseline” dataset (positive by the formol ether concentration method, with no other *Ascaris*, *Necator* or *Ancylostoma* infection as determined by RT-qPCR and egg counting (Rosa et al., 2018), and no evidence of protozoan parasites observed by the formalin ether concentration test; N = 20 infected and N = 47 uninfected). Only samples from individuals treated with albendazole (400 mg) who cleared the infection over the course of a 2 year treatment (every 3 months; eight total treatments) were used in the “dewormed” cohort (N = 11). This repeated treatment regime is necessary to effectively clear *Trichuris* infection (Vlaminck et al., 2020). The formol ether method is semi-quantitative, so before treatment, samples had moderate to high infection intensities. Additional human samples from this dataset were included as control samples for (i) uninfected and untreated (N=16) and (ii) uninfected and albendazole-treated (N=16). Gender and age metadata for individuals from each sample cohort are summarized in **Table 1**.

**Table 1:** Gender and age metadata summary for human samples used in each comparison.

Statistic		Primary comparisons			Control comparisons	
		Baseline Uninfected	Baseline Infected	Deworming	Uninfected, no treatment	Uninfected, anthelmintic treatment
Gender (# of individuals)	Female	24	14	8	10	9
	Male	23	6	3	6	7
Age (years)	Lowest	4.2	4.7	5.1	5.0	4.9
	Highest	55.4	57.9	57.9	50.4	50.4
	Average	29.4	19.5	24.2	34.6	28.6

### 16S rRNA Gene Sample Sequencing and Analysis

**Figure 1:**
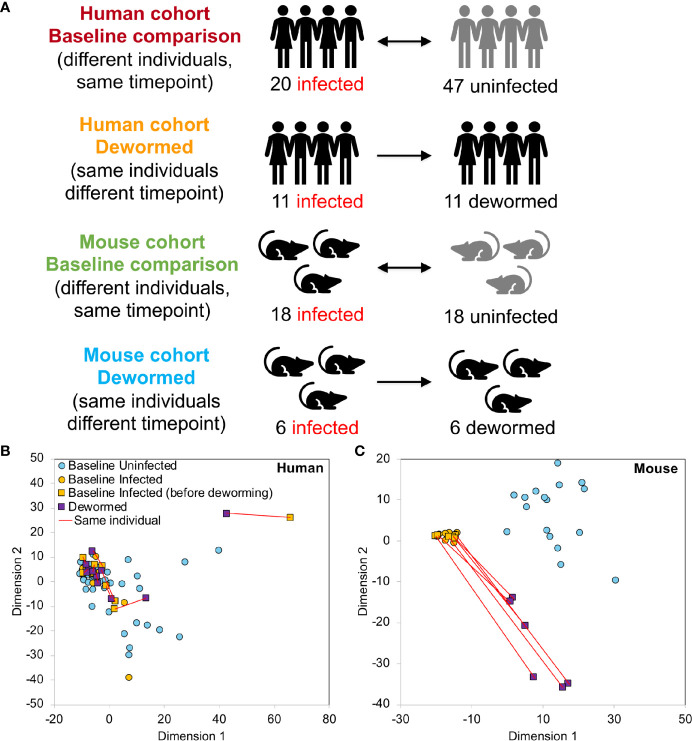
Overview of 16S rRNA gene sample sets. **(A)** Sample cohorts used for differential intestinal microbiome analysis comparing samples from *Trichuris trichiura*-infected and uninfected humans and *T. muris*-infected and uninfected mice before and after deworming. At “baseline”, the microbiomes of different individuals were compared and in the “dewormed” comparisons, the microbiomes of same individuals before and after curing of the infection were compared. MDS-based clustering of samples based on their normalized microbiome abundance profiles are shown for human **(B)** and mouse **(C)**. Red lines connect samples from the same individuals before and after deworming. Normalization was performed by dividing OTU read counts by the total number of read counts assigned to OTUs in each sample.

The V1-V2 hypervariable region of the 16S rRNA gene was amplified by PCR (30 cycles) using forward and reverse primers “AGAGTTTGATCMTGGCTCAG” and “CTGCTGCCTYCCGTA” (respectively). Samples were assessed post amplification on the LabChip GXII (Caliper Life Sciences, Hopkington, Massachusets). 5nM dilutions of amplified samples were prepared and pooled for qPCR. PCR products were purified and sequenced on the MiSeq Genome Sequencer (Illumina, San Diego, California). Illumina paired reads were assembled using FLASH (v1.2.7) (Magoc and Salzberg, 2011) and low quality and chimeric sequences were removed using the UCHIME *de novo* tool (Edgar et al., 2011) from inside the Mothur package (v 1.37.5-64) (Schloss et al., 2009). The processed V1-V2 amplicons were clustered into OTUs (99% similarity for strain-level clustering) and classified using the developer’s QIIME2 (Bolyen et al., 2019) docker container (qiime2/core:2018.8), using a classifier based on SILVA (release 132) (Quast et al., 2013), a comprehensive database that provides accurate annotations (Balvociute and Huson, 2017). 16S rRNA gene read sequences can be downloaded from SRA (BioProject PRJNA679627). Read counts were normalized per sample by dividing the number of reads associated with each OTU by the total number of reads assigned to any OTU. The taxonomic identifications used throughout the manuscript are the ones provided by SILVA (Quast et al., 2013). Complete OTU taxonomy, counts and relative abundance values per sample are provided in **Supplementary Table S1A–C**. The human and mouse samples generated during this study were statistically compared in four comparisons (**Figure 1A**), each of which compared infected to uninfected individuals. For each host species, samples were compared at “baseline” (different individuals at the same timepoint, either infected only with whipworm, or not infected with any of the three main STH species) or they were compared before and after deworming with anthelmintic treatment (the same individuals at different time points). Control comparisons were performed between the first timepoint (corresponding to infected individuals at baseline) and the second timepoint (corresponding to dewormed individuals following anthelmintic treatment) and included uninfected individuals receiving no treatment (both species), uninfected individuals receiving anthelmintic treatment (both species) and infected individuals receiving no treatment (mouse only) (**Supplementary Figure S1**).

LEfSe (Linear discriminant analysis Effect Size) (Segata et al., 2011) was used for differential taxa abundance testing, using default recommended settings according to the author’s instructions, at an adjusted P ≤ 0.05 for significance and requiring an LDA effect size of at least 2 in order to identify differentially abundant taxa. LEfSe’s algorithm performs class comparison tests, validates for biological consistency, and considers the hierarchy of the taxonomy to perform tests at all taxonomic levels. We have previously used this statistical approach to identify common helminth-associated taxa between human cohorts (for any of the three main STH species) (Rosa et al., 2018), and it has also been used to identify intestinal bacteria associated with pathogenic infections (Villarino et al., 2016), to identify intestinal biomarkers for disease (Segata et al., 2011), and to track microbiome recovery following disease (Rooks et al., 2014). Complete LEfSe statistics for each comparison are provided in **Supplementary Table S1D**.

Shannon index diversity values were calculated for each sample using the normalized read counts across all taxa using the “diversity” function in the “vegan” library in R (version 2.5), and Bray-Curtis dissimilarity values were calculated using the “vegdist” function. Multidimensional scaling (MDS) was performed using the same Bray-Curtis dissimilarity values, using the R function “cmdscale” (R version 4.0.2). Clustering with t-SNE was also performed using the “Rtsne” package in R (version 0.15) using the same Bray-Curtis dissimilarity values, in addition to Non-metric Multidimensional Scaling (NMDS) using the “bestnmds” function in the “LabDSV” package (version 2.0; http://ecology.msu.montana.edu/labdsv/R/). One-way analysis of variance (ANOVA) testing was performed with a Tukey HSD post-hoc test, for both the Shannon diversity and Bray-Curtis dissimilarity comparisons.

### RT-qPCR Analysis

Relative abundance of selected infection-associated taxa was measured by real-time quantitative PCR (RT-RT-qPCR) for the mouse samples and for a subset of the human samples from the 16S rRNA gene analysis for which sufficient template DNA was still available (nine individuals before and after deworming). All consumables were purchased from ThermoFisher Scientific (Waltham, MA, USA), unless otherwise specified. Reactions for *Blautia* were performed in a 25 µl set-up per sample using Power SYBR Green Master Mix, and a final concentration of 300 nM forward and reverse primers with 2 µl of stool DNA extract was run through 40 amplification cycles according to the standard master mix manufacturer’s two-step protocol. *Lachnospiracea*, *Prevotella*, and *Collinsella* were detected and quantified using Taq Man Fast Advanced Mastermix in a 20 µl set-up. Both primers were used in a final reaction concentration of 400 nM and the concentration of the probe was 125 nM. Two microliters of stool DNA extract was used per reaction. All reactions were performed using a QuantStudio 6 Flex Real-Time PCR System. The cycling conditions were as follows: A pre-read stage of 30 s at 60°C, followed by 20 s at 95°C. The 40 cycles amplification consisted of a 1 s denaturation step at 95°C followed by 20 s annealing and extension at 60°C. The Post-Read stage is 30 s at 60°C. Each DNA sample was tested in duplicate against genus-specific and universal bacterial 16S rRNA gene primers (400 nM) (**Supplementary Table S2**). Cycle threshold (CT) values were calculated by taking the average CT for each sample and primer set. Relative fold change in bacterial abundance was calculated using the 2^–ΔΔCT^ method using the 16S rRNA gene CT values as a calibrator. P values were calculated from the Δ CT values comparing before and after deworming samples using a two-tailed T test with unequal variance (Schmittgen and Livak, 2008).

## Results and Discussion

Previous studies of associations among microbiome and whipworm infections have been performed in a single host species. To support translational discoveries, a comprehensive identification of common bacterial taxa protective from (and supportive of) infection across hosts is needed. Therefore, we studied microbiome responses to whipworm infection in a murine model and compared it to a human cohort from Indonesia from our previous study (Rosa et al., 2018). From the human dataset, we re-analyzed only those samples which were infected with *T. trichiura* and no other nematode species (N = 20 infected and N = 47 uninfected at baseline; N = 11 infected and dewormed with albendazole), so that we could provide the closest possible comparison to the *T. muris*-infected C57BL/6N/STAT6KO mice analyzed (N = 18 infected and N = 18 uninfected at baseline; N = 6 infected and dewormed). This mouse strain provides a relatively high and uniform worm burden throughout the treatment groups, and modulation of STAT6 is a predictor of resistance to gastrointestinal nematodes including *Trichuris* species in humans, pigs and “rewilded” mice that are affected by changes in the intestinal microbiome (Moller et al., 2007; Leung et al., 2018; Dawson et al., 2020). The human and mouse samples were analyzed using same pipeline and then statistically compared in four comparisons (**Figure 1A**). For each host species, samples were compared at “baseline” (different individuals at the same timepoint, either infected only with whipworm, or not infected with any helminth) or they were compared before and after curing whipworm infection (the same individuals at different timepoints).

Control comparisons across the deworming timepoints were also included (**Supplementary Figure S1**) for uninfected humans receiving no anthelmintic treatment (N=16), uninfected humans receiving albendazole treatment (N= 16), infected mice receiving no anthelmintic treatment (N=6), uninfected mice receiving no anthelmintic treatment (N=6), and uninfected mice receiving mebendazole/ivermectin treatment (N= 6).

### Microbiome Profile Overview and Analysis

MDS-based clustering of samples showed that while the overall human microbiome profiles did not have any observable correlation with whipworm infection status (**Figure 1B**), the mouse samples clustered separately by infection status (**Figure 1C**), with the dewormed samples clustering between the infected and uninfected samples. This pattern in microbiome profiles was expected since human samples have high variability due to differences in genders, ages, village locations, diets, and histories of infection status, while the mice were in a laboratory-controlled environment that ensured consistent microbiomes and therefore more consistent responses to infection. Similar clustering patterns were also observed with both NMDS and tSNE-based clustering (**Supplementary Figure S2A**), and human data did not cluster according to either gender or age (**Supplementary Figure S2B**). Control sample cohorts showed similar clustering profiles, with infected mouse samples clustering away from uninfected samples, regardless of timepoint or anthelmintic treatment (**Supplementary Figure S3**). Overall microbiome profiles in each sample for both human and mouse cohorts are summarized at the class level in **Supplementary Figure S4**.

**Figure 2:**
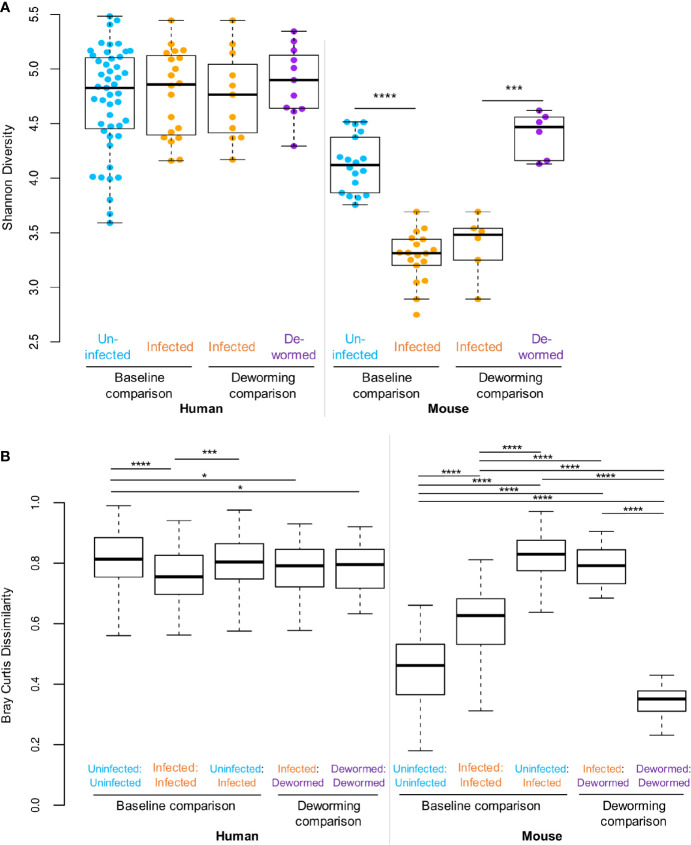
Statistical comparisons of diversity measures. **(A)** Shannon (alpha) diversity values for all samples in each sample set. **(B)** Bray-Curtis dissimilarity (beta diversity) values either between samples from the same sets, or between sample sets. *P ≤ 0.05, ***P ≤ 0.001, ****P ≤ 0.0001, according to ANOVA with Tukey HSD *post-hoc* test. F-statistic values for human and mouse were 13.8 and 464.6, respectively (P < 10^−5^ in both cases).

The Shannon diversity index (**Figure 2A**) represents the within-sample or “alpha” diversity, reflecting the number and the evenness of the distribution of microbiome members. While there were no significant differences between infection groups in the human cohort, the within-sample diversity was significantly lower (P < 10^−10^, two-tailed T-test, unequal variance) in the whipworm-infected mice at baseline compared to uninfected mice, and this diversity was increased back to the same level as the uninfected samples following deworming (P < 10^−10^). Bray-Curtis dissimilarity (**Figure 2B**) was used to quantify the between-sample differences in the overall microbiome profiles. The samples from infected humans had microbiome profiles that were significantly more similar to each other compared to samples from uninfected humans, or compared to dewormed subjects (P < 10^−10^). However, in the more controlled laboratory setting, uninfected samples had significantly lower baseline diversity that was disrupted by whipworm infection, resulting in higher average dissimilarity between infected samples and uninfected samples, compared to within-infected and within-uninfected sample groups (P < 10^−10^). Following deworming, the between-sample differences were very low in the mice, having lower diversity than between infected samples (P < 10^−10^), and even lower diversity than between baseline uninfected samples (P = 1×10^−5^). These results suggest that whipworm-associated disruptions in the microbiomes resolved in similar ways in the dewormed samples. Relative abundance values per OTU per sample can be accessed in **Supplementary Table S1C**.

**Figure 3:**
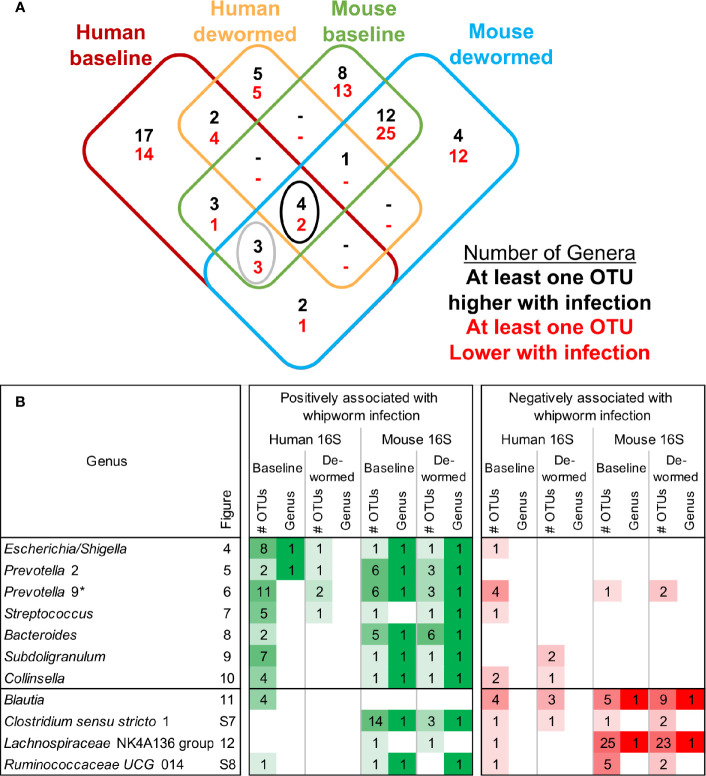
Overview of differential taxa abundance results. **(A)** Venn diagram of differentially abundant genera counts across each comparison (corresponding to each of the uninfected vs infected comparisons outlined in **Figure 1A**), requiring at least one OTU belonging to the genera to be signficantly differentially abundant. Black circle = differentially abundant genera in each of the four comparisons, gray circle = differentially abundant genera in the human baseline, mouse baseline, and mouse dewormed comparisons, but not in the human dewormed comparison. **(B)** Genera from the circled regions of panel **(A)**, indicating the genera name, the corresponding figure number displaying full OTU abundance data, the number of OTUs significantly associated with whipworm infection, and whether the entire genus was significantly associated with infection. Lighter and darker shades of green and red are used to indicate lower/higher significant OTU counts (respectively) for each genus. *Prevotella 9 was also one of the three genera downregulated in three comparisons (human baseline, mouse baseline and mouse dewormed comparisons, but not in the human dewormed comparison).

Using LEfSe (Segata et al., 2011), we identified significant differentially abundant microbiome taxa at all taxonomic levels in each of the four comparisons (baseline infected vs uninfected, and infected vs dewormed, in both humans and mice; **Figure 1A**). The number of significant differentially abundant OTUs is shown in **Table 2** and **Supplementary Figure S5A**. Although none of the significant differentially abundant OTUs overlapped between the two host species, these OTUs represented variable 16S rRNA gene sequences from many overlapping species, genera, families, orders, classes, and phyla (**Supplementary Figure S5B–G** **;** **Supplementary Table S1D**). For the sake of simplifying the analysis of these comprehensive taxonomic results, we focused on the genera-level annotations of the significant OTUs. Genera were identified that were either significantly differentially abundant at the entire genera level, as well as those that were not significant but were represented by one or more OTUs that were significant in the comparison (**Figure 3A**). The four comparisons shown in **Figure 3A** correspond to the comparisons shown in **Figure 1A**, and the black-circled genera represent genera with at least one significant OTU in each of the four comparisons while the grey-circled genera represent genera with at least one significant OTU in each of the comparisons, excluding the dewormed human cohort, since this utilized a smaller sample set (N = 11) with a high noise background and weaker statistical signal. Each of these genera is discussed in detail below.

**Table 2:** Summary of sample groups used for each statistical comparison, and the counts of the number of significant OTUs and taxa at any taxonomic level, based on LEfSe results.

Comparison group		Species	Samples in Group 1		Samples in Group 2		# OTUs significantly higher		Total # taxa significantly higher	
			Status	N	Status	N	Group 1	Group 2	Group 1	Group 2
Four primary comparisons	Baseline Comparison	Human	Uninfected	47	Infected	20	43	88	58	116
		Mouse	Uninfected	18	Infected	18	257	107	373	169
	Deworming Comparison (paired)	Human	Infected	11	Dewormed	11	23	18	39	18
		Mouse	Infected	6	Dewormed	6	69	309	125	395
Control comparisons (all paired)	Uninfected, no treatment	Human	Before	16	After	16	1	0	5	0
		Mouse	Before	6	After	6	20	47	27	71
	Uninfected, anthelmintic treated	Human	Before	16	After	16	0	0	0	0
		Mouse	Before	6	After	6	41	56	59	90
	Infected, untreated	Mouse	Before	6	After	6	5	20	9	27

We have also included control LEfSe comparisons including (i) uninfected, untreated (humans and mice), (ii) uninfected, treated (humans and mice), and (iii) infected untreated (mice only) (**Supplementary Figure S1**). Overall results for these are shown in **Table 2**, complete LEfSe results for these five comparisons are shown in **Supplementary Table S1D**, and any overlaps between OTUs in control comparisons and the genera from the four primary comparisons will be highlighted in the text below.

### Microbiome Taxa Associated With Infection Across Human and Mice

**Figure 4:**
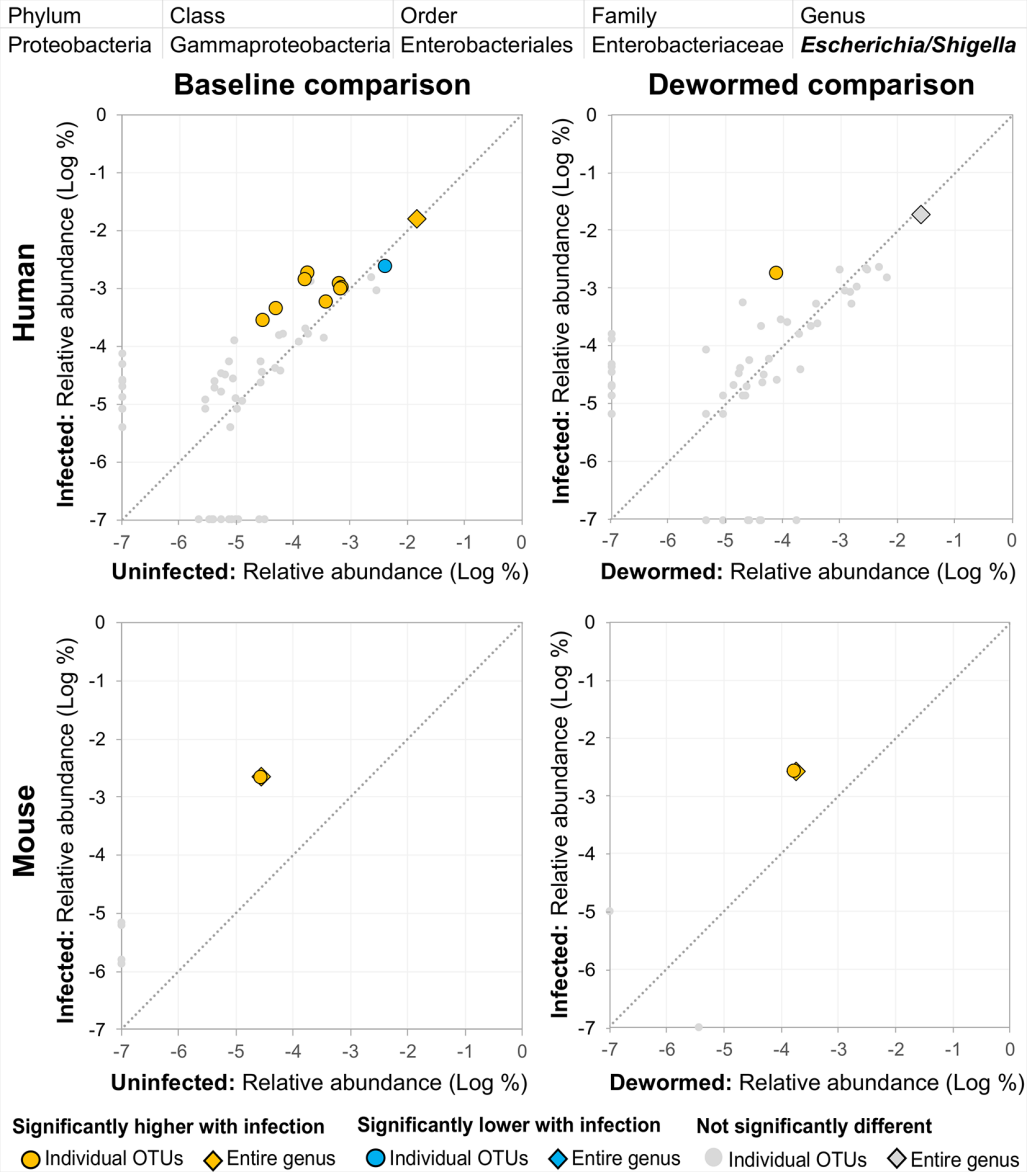
The relative abundance (%) of all *Escherichia/Shigella* (genus) OTUs in each differential comparison among samples from whipworm-infected and uninfected humans and mice. Significant differentially abundant OTUs in each comparison (as identified by LEfSe) are colored in orange (higher with infection) and blue (lower with infection). The sum of all OTUs in the genus is represented with a diamond symbol. This is one of four genera with at least one OTU significantly higher in infection in all four comparisons.

**Figure 5:**
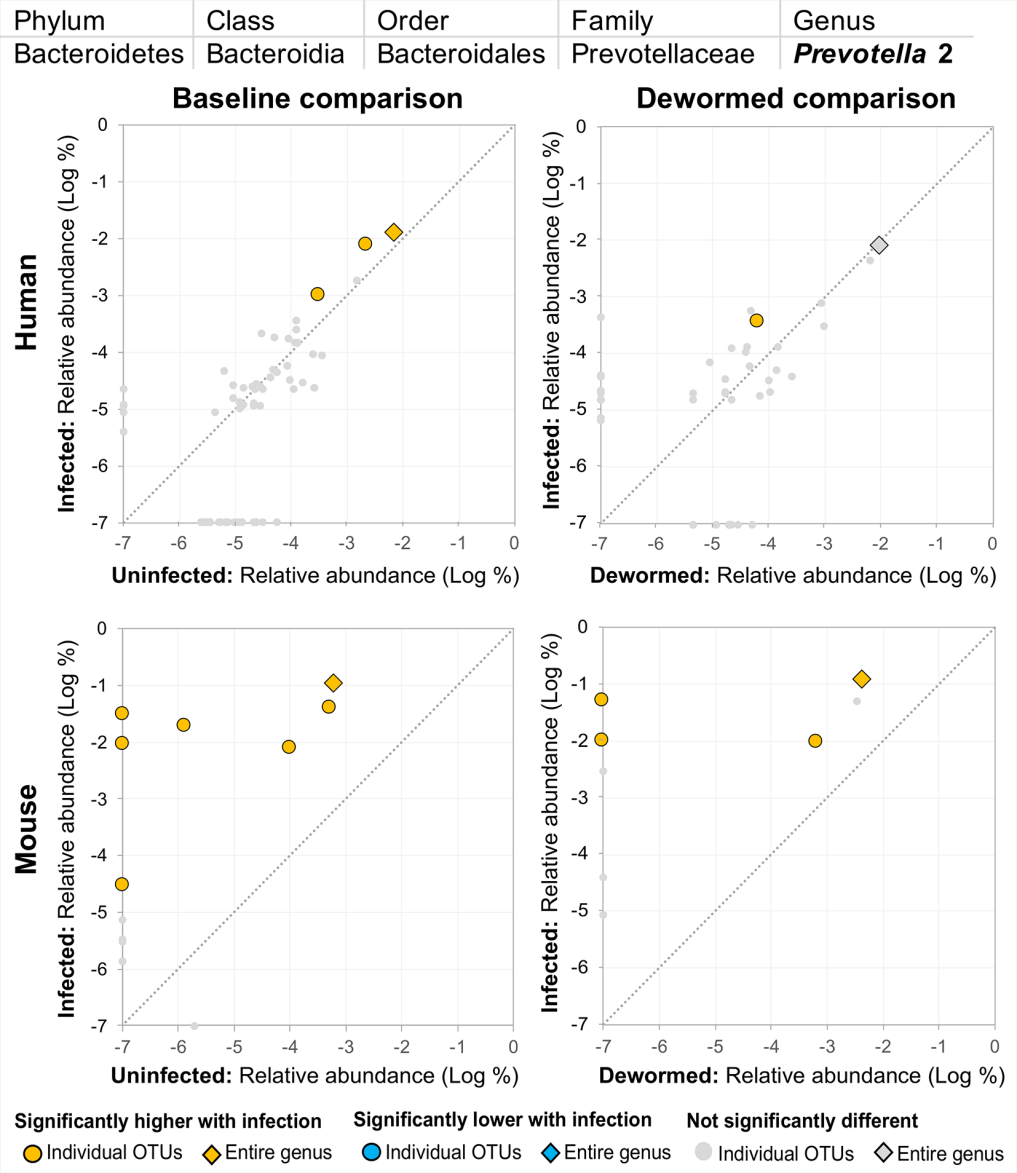
The relative abundance (%) of all *Prevotella* 2 (genus) OTUs in each differential comparison among samples from whipworm-infected and uninfected humans and mice. Significant differentially abundant OTUs in each comparison (as identified by LEfSe) are colored in orange (higher with infection) and blue (lower with infection). The sum of all OTUs in the genus is represented with a diamond symbol. This is one of four genera with at least one OTU significantly higher in infection in all four comparisons.

**Figure 6:**
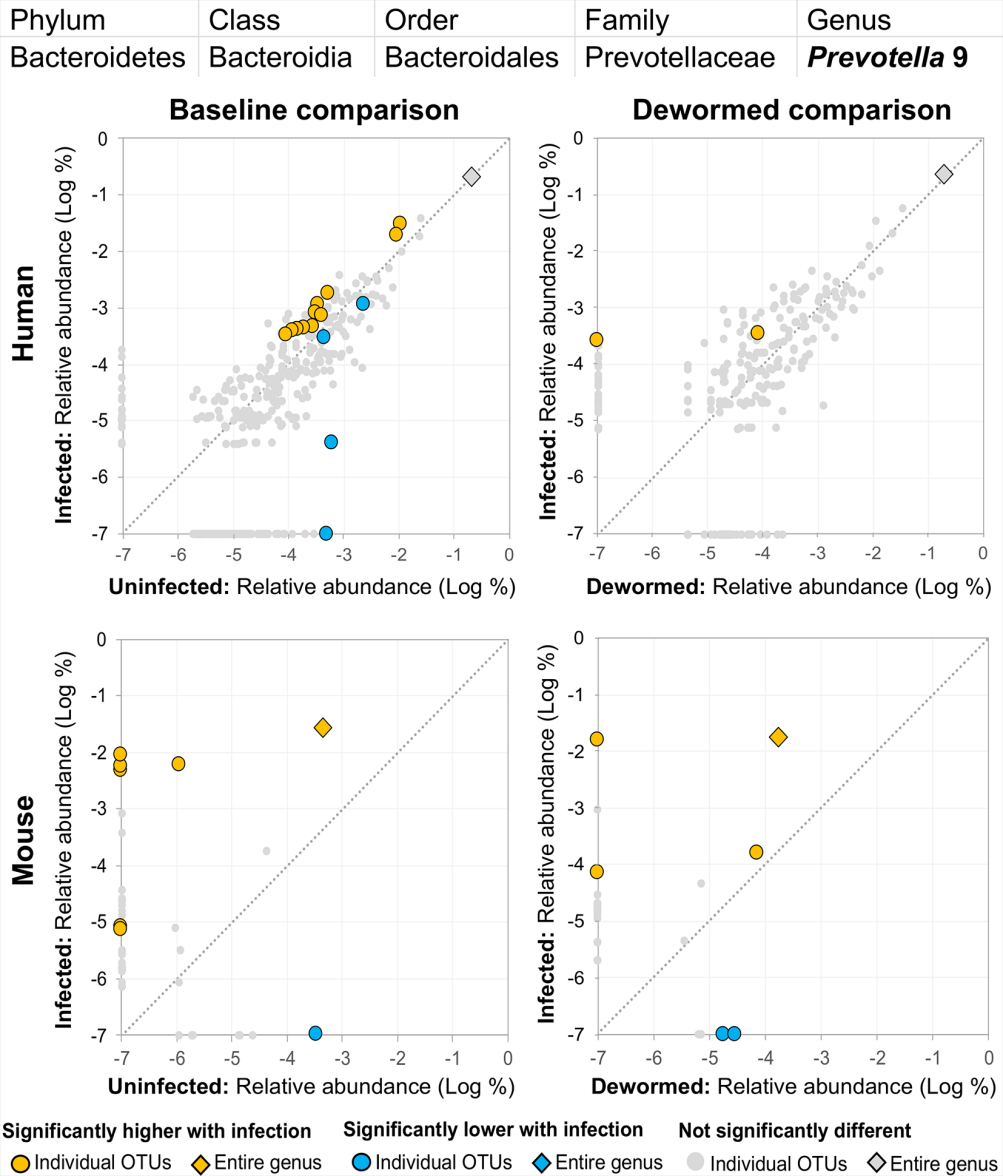
The relative abundance (%) of all *Prevotella* 9 (genus) OTUs in each differential comparison among samples from whipworm-infected and uninfected humans and mice. Significant differentially abundant OTUs in each comparison (as identified by LEfSe) are colored in orange (higher with infection) and blue (lower with infection). The sum of all OTUs in the genus is represented with a diamond symbol. This is one of four genera with at least one OTU significantly higher in infection in all four comparisons and is also one of three genera with at least one OTU significantly lower in infection in three of four comparisons (because different OTUs were both higher and lower in the same comparisons).

**Figure 7:**
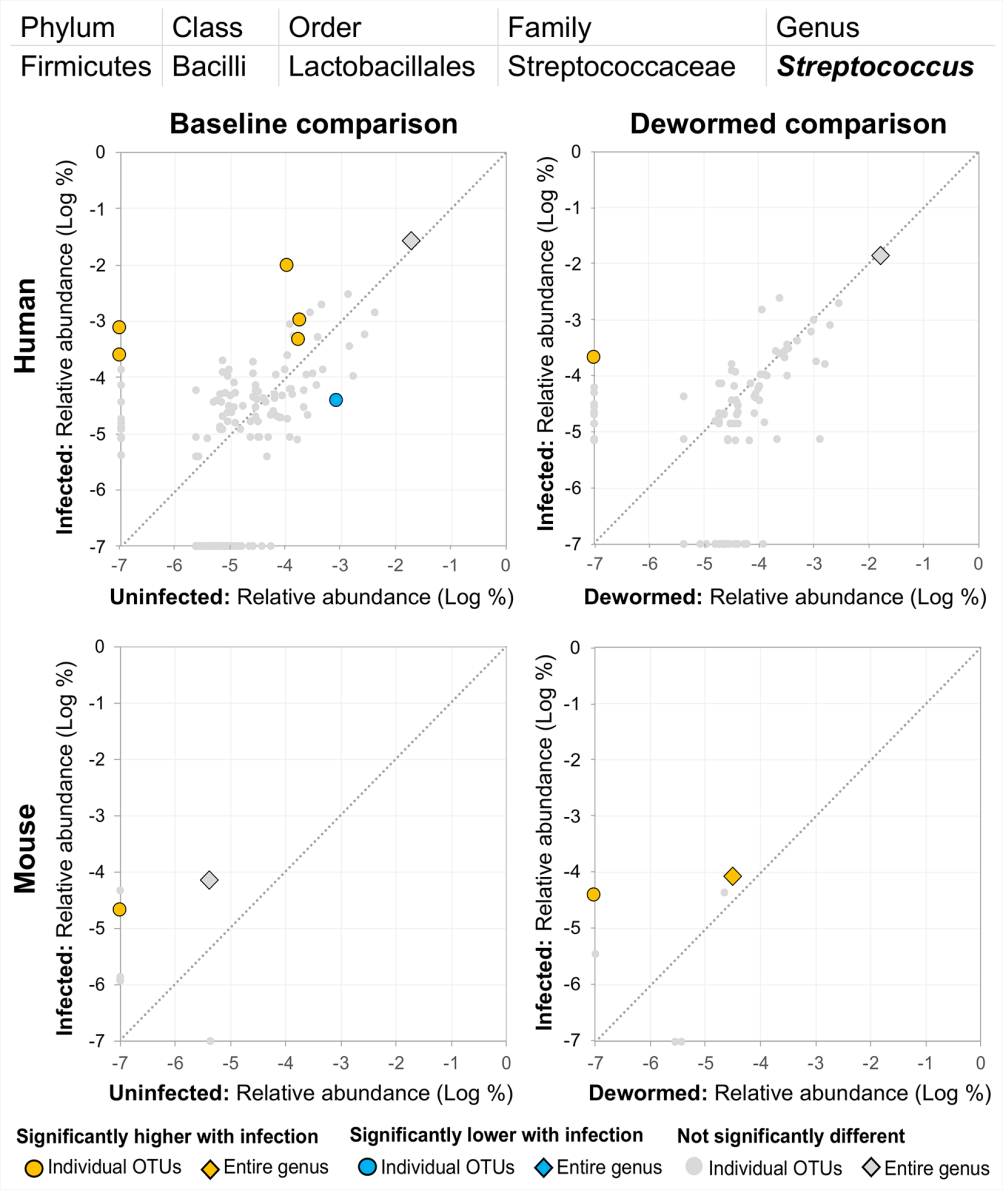
The relative abundance (%) of all *Streptococcus* (genus) OTUs in each differential comparison among samples from whipworm-infected and uninfected humans and mice. Significant differentially abundant OTUs in each comparison (as identified by LEfSe) are colored in orange (higher with infection) and blue (lower with infection). The sum of all OTUs in the genus is represented with a diamond symbol. This is one of four genera with at least one OTU significantly higher in infection in all four comparisons.

Based on the results from the LEfSe analysis, four bacterial genera were represented by OTUs that were significantly more abundant in the samples of infected humans and mice compared to uninfected individuals, and which were less abundant following deworming (positively associated with infection). These included:

(i) *Escherichia/Shigella* (**Figure 4**) was represented by a single OTU in mice (species *Escherichia coli* Xuzhou21), but several OTUs in human. In a previous mouse whipworm infection study, *Escherichia* was one of just four genera significantly associated with infection (200-egg infection in Swiss Webster outbred mice, 45 days post-infection) (Schachter et al., 2020), and in another, *Escherichia* was only detectable following infection (20-egg infection, C57BL/6 mice, 35 days post-infection) (Holm et al., 2015). Here, we observed that it not only increased with infection in both mice and humans (at both the OTU and genera level), but also decreased following deworming in mice (OTU and genera levels) and humans (OTU level). In control uninfected anthelminthic-treated mice over the same time period, this genus and the same OTU significantly increased over time (the opposite of the pattern observed with deworming). This finding is particularly interesting since *E. coli* (spanning many different strains) can induce *T. muris* egg hatching *in vitro* (Vejzagic et al., 2015), so known associations exist between this bacterial genus and whipworm.
(ii) *Prevotella* 2 (**Figure 5**) and (iii) *Prevotella* 9 (**Figure 6**) were higher in whipworm-infected compared to uninfected individuals. For both *Prevotella* 2 and *Prevotella* 9, the entire genus was significantly associated with infection in both comparisons in mice, and for *Prevotella* 2, the baseline comparison in the humans was also significant for the entire genera. In both genera, there were several representative OTUs in each of the mouse comparisons which had zero detection in the sample from uninfected subjects but high detection in the samples from infected subjects, indicating a potential presence/absence relationship with whipworm infection (although low levels may be present below the detection limit of the experiment). In control samples, three OTUs and the entire genus for *Prevotella* 2 were significantly increased in uninfected treated mice over the testing time period (the opposite of the pattern observed in infected samples over the time period). In the same control comparison, one OTU for *Prevotella* 9 was significantly increased over the time period, and the entire genus was increased (but not signficantly). *Prevotella* was identified as significantly more abundant in a previous mouse infection study with the small intestinal parasitic nematode *H. polygyrus bakeri* (200 iL3 infection in C57BL/6 mice, 14 days post-infection) (Rausch et al., 2013), but has also been observed to decrease in whipworm*-*infected C57BL/6 mice (41 days post-infection) (Houlden et al., 2015). However, unlike the current study which used a short-term high-infection model, this previous study (Houlden et al., 2015) used a chronic low-burden infection model, which may have caused some of the disagreement for this particular genus. In humans, *Prevotella* was significantly increased in helminth-infected (*Ascaris* and/or whipworm) individuals from Colombia (Toro-Londono et al., 2019). Prevotella represents one of the two enterotypes of the human intestinal microbiome (the other being *Bacteroides*) (Cheng and Ning, 2019), and it often dominates other genera in abundance, so significant shifts in its abundance may reflect overall disturbances in microbiome profiles induced by the whipworm.
(iv) *Streptococcus* (**Figure 7**) was represented by at least one significant OTU that was detected in samples from infected but not uninfected subjects in each of the four comparisons. In a previous study of whipworm-infected children from Ecuador, 10 of 50 infected children had microbiomes dominated by unusually high *Streptococcus*, which is not typically dominant in the intestines of healthy individuals (Cooper et al., 2013).

**Figure 8:**
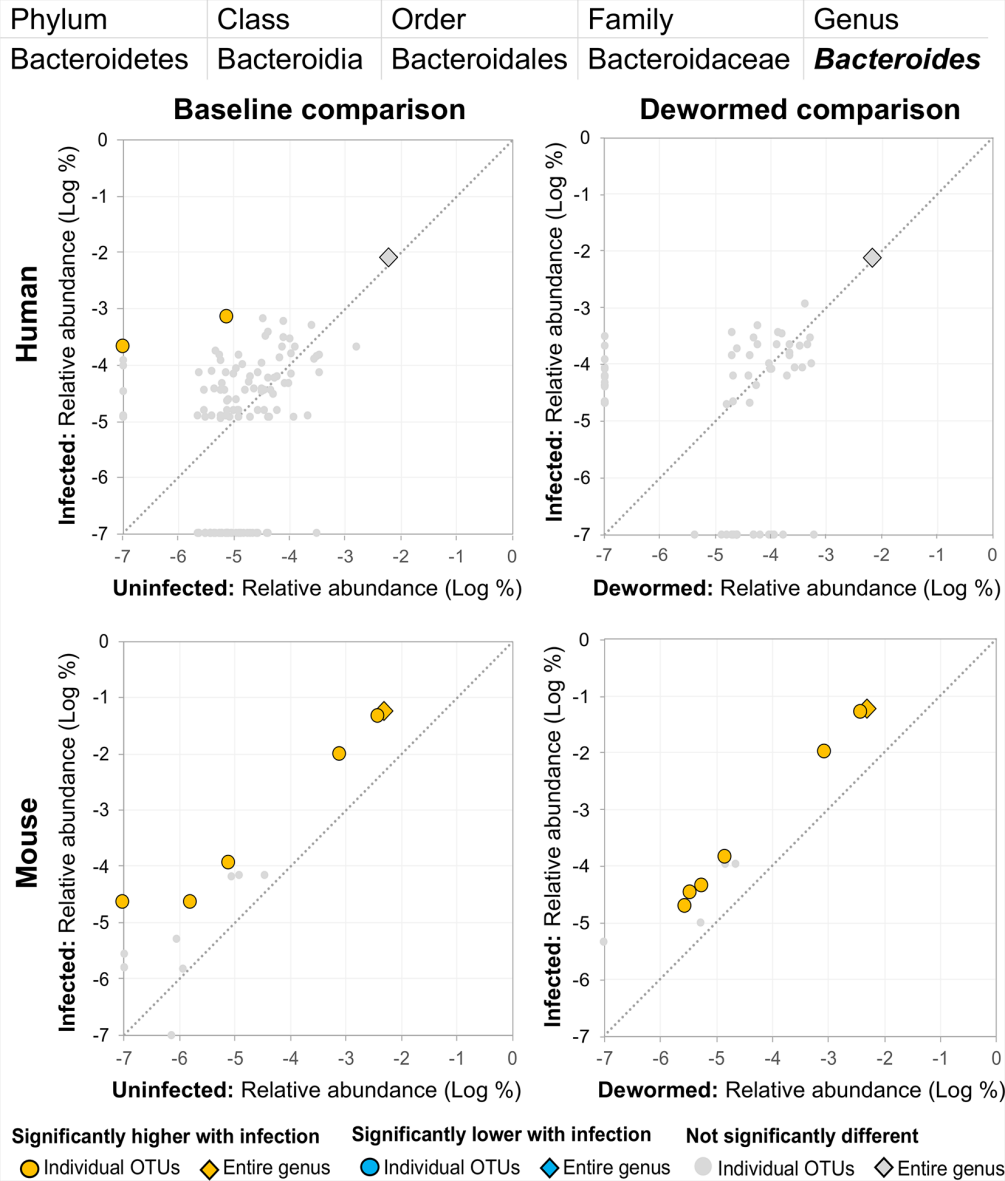
The relative abundance (%) of all *Bacteroides* (genus) OTUs in each differential comparison among samples from whipworm-infected and uninfected humans and mice. Significant differentially abundant OTUs in each comparison (as identified by LEfSe) are colored in orange (higher with infection) and blue (lower with infection). The sum of all OTUs in the genus is represented with a diamond symbol. This is one of three genera with at least one OTU significantly higher in infection in three of four comparisons.

**Figure 9:**
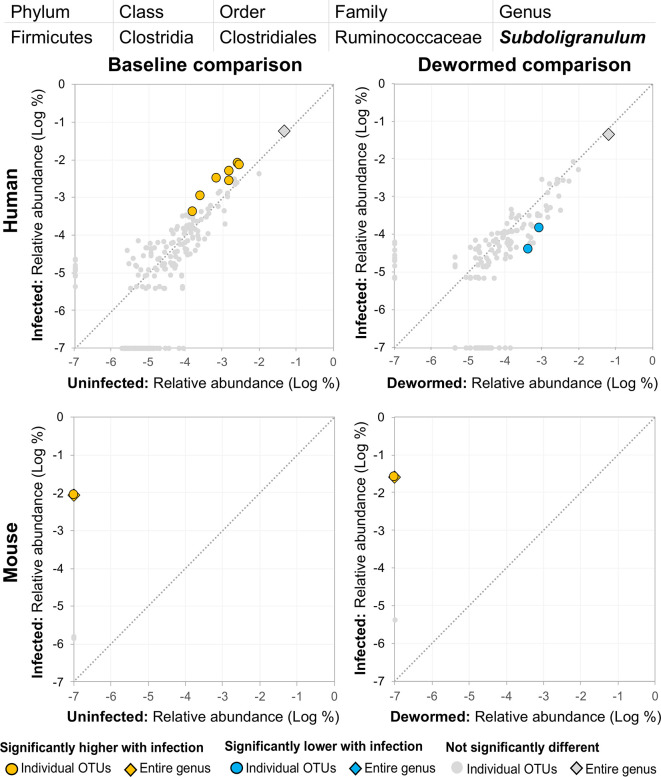
The relative abundance (%) of all *Subdoligranulum* (genus) OTUs in each differential comparison among samples from whipworm-infected and uninfected humans and mice. Significant differentially abundant OTUs in each comparison (as identified by LEfSe) are colored in orange (higher with infection) and blue (lower with infection). The sum of all OTUs in the genus is represented with a diamond symbol. This is one of three genera with at least one OTU significantly higher in infection in three of four comparisons.

**Figure 10:**
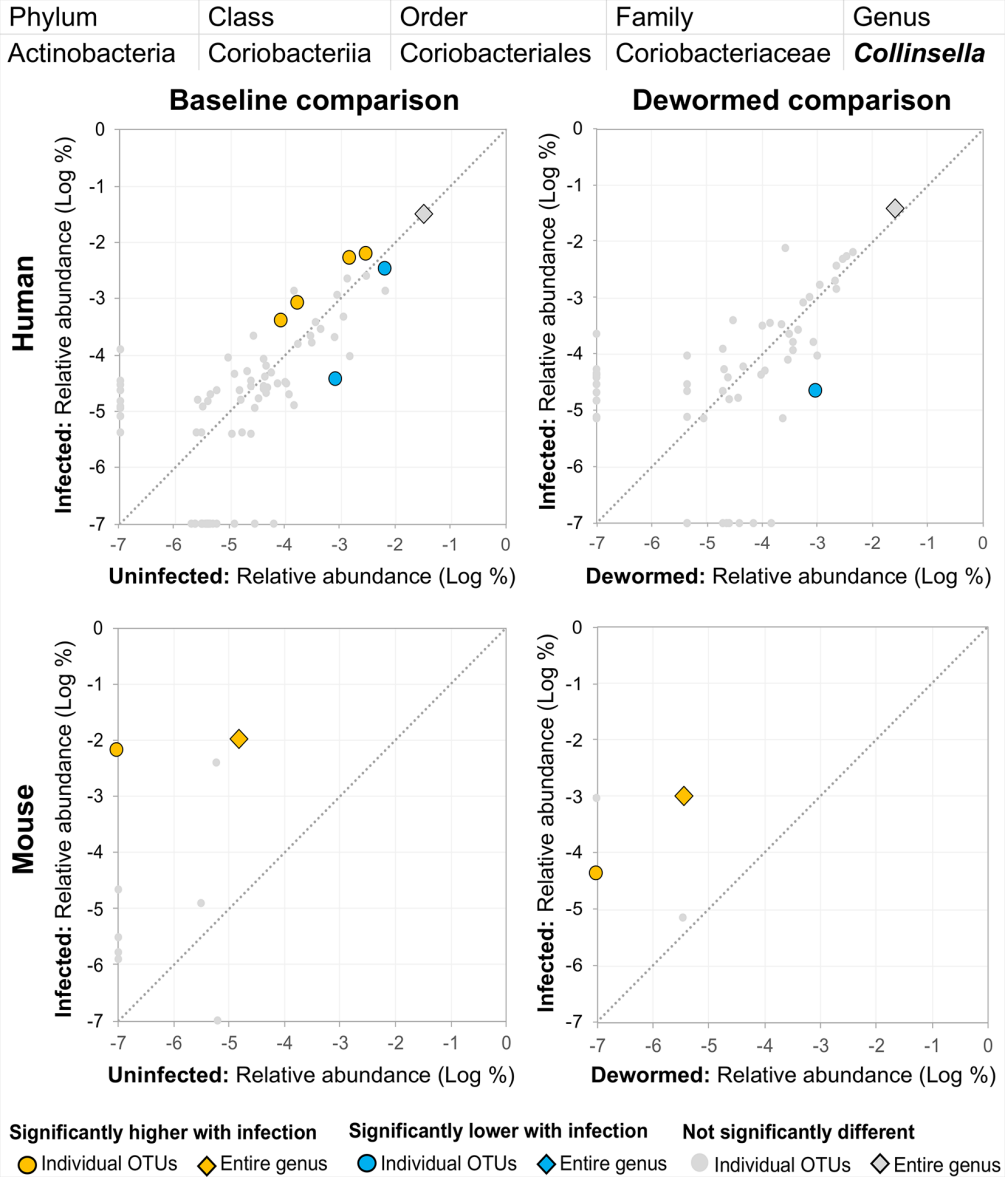
The relative abundance (%) of all *Collinsella* (genus) OTUs in each differential comparison among samples from whipworm-infected and uninfected humans and mice. Significant differentially abundant OTUs in each comparison (as identified by LEfSe) are colored in orange (higher with infection) and blue (lower with infection). The sum of all OTUs in the genus is represented with a diamond symbol. This is one of three genera with at least one OTU significantly higher in infection in three of four comparisons.

Three bacterial genera were represented by OTUs that were significantly more abundant in the intestines of infected humans and mice compared to uninfected individuals, and that were less abundant following deworming (positively associated with infection) in mice, but not in humans. These included:

(i) *Bacteroides* (**Figure 8**), for which many OTUs and the entire genus were significantly more abundant in the mice. In a previous mouse whipworm infection experiment, uninfected mice had undetectable levels of *Bacteroides*, but infected mice exhibited large amounts (200-egg infection in Swiss Webster outbred mice, 45 days post-infection) (Schachter et al., 2020), and *Bacteroides* is one of the major components of the *T. muris* microbiome (White et al., 2018). Here, we also identified two *Bacteroides* OTUs that were also significantly higher in whipworm-infected humans. Just one of the *Bacteroides* OTUs significant in the mice was also significant in one of the control comparisons (infected, untreated mice).
(ii) *Subdoligranulum* (**Figure 9**) was represented almost entirely by a single highly abundant OTU in infected but not uninfected mouse samples at baseline or after deworming, while seven relatively highly abundant OTUs were associated with infection at baseline in human. We previously associated *Subdoligranulum* with eventual self-clearing of helminth infections in samples from an Indonesia cohort (considering all three STH infections) (Rosa et al., 2018), but not otherwise associated with parasitic infections.
(iii) *Collinsella* (**Figure 10**) was significantly higher in both comparisons in mice, along with one significant OTU which was only detectable in samples from infected humans. We had also previously identified *Collinsella* as being associated with general helminth infection (inclusive of whipworm, *Ascaris*, and hookworm) in a human cohort from Liberia (Rosa et al., 2018), but it has not been associated with whipworm infection in other studies.

Overall, we consistently identified seven bacterial genera with representative OTUs that are significantly associated with whipworm infection, most of which have been identified in previous literature, and also demonstrated genera-level significant associations in both human and mouse host species (*Escherichia* and *Prevotella* 2 at baseline in humans, and all genera at both baseline and following deworming in mice, with the single exception of *Streptococcus* at baseline; **Figure 3B**). Most notably, we identified *Escherichia* as being positively associated in all four comparisons (baseline infected vs uninfected, and infected vs dewormed, in both human and mice), a genus which has known for functional interactions with whipworm.

### Microbiome Taxa Negatively Associated With Infection

**Figure 11:**
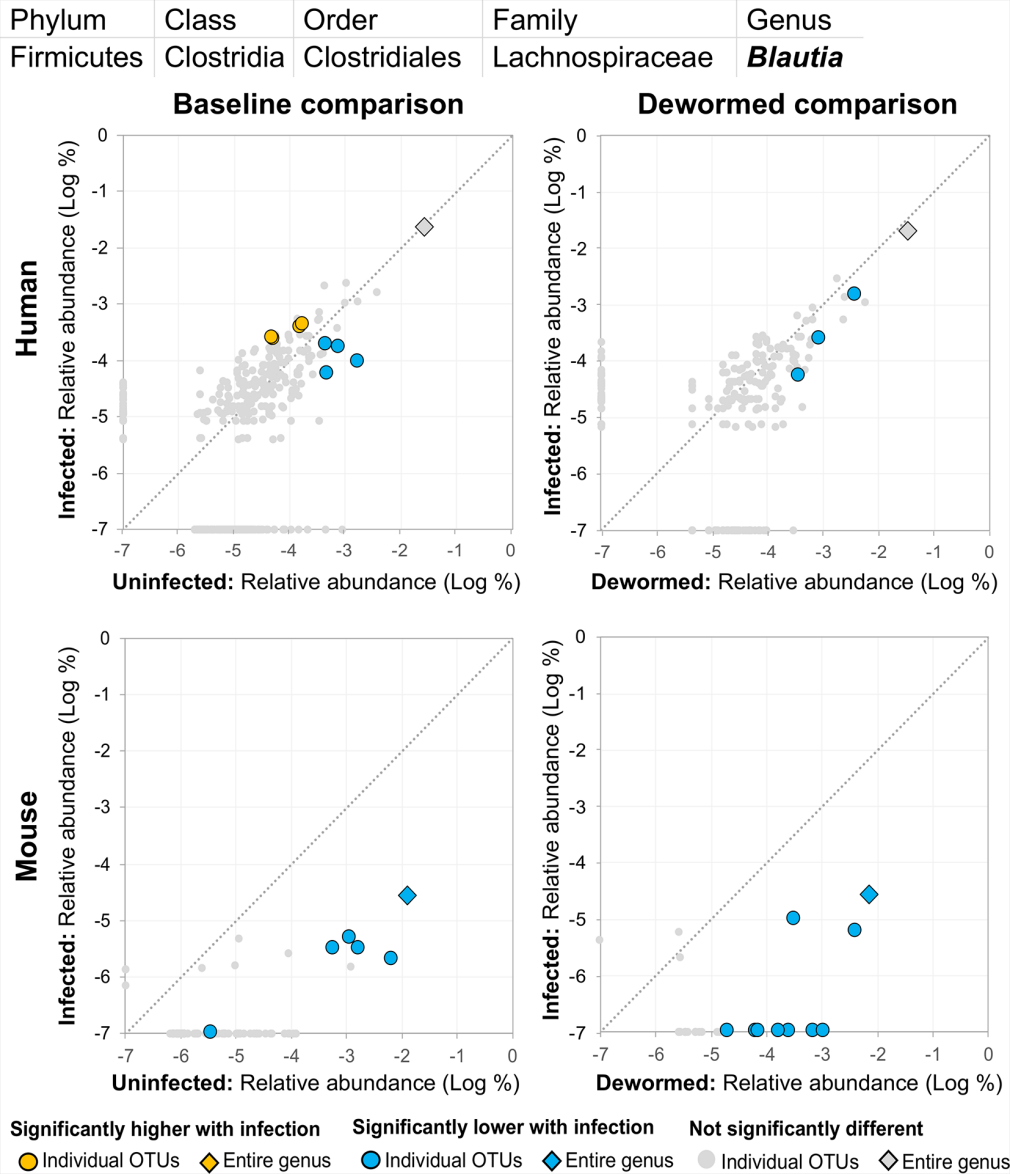
The relative abundance (%) of all *Blautia* (genus) OTUs in each differential comparison among samples from whipworm-infected and uninfected humans and mice. Significant differentially abundant OTUs in each comparison (as identified by LEfSe) are colored in orange (higher with infection) and blue (lower with infection). The sum of all OTUs in the genus is represented with a diamond symbol. This is one of two genera with at least one OTU significantly lower in infection in all four comparisons.

Two bacterial genera were represented by OTUs that were significantly less abundant in the samples of infected humans and mice, and which were more abundant following deworming (negatively associated with infection):

(i) *Blautia* (**Figure 11**), a member of the *Lachnospiraceae* family, was previously identified in a study of whipworm (*T. suis*) infection in pigs as one of the intestinal microbiome genera most significantly lower during infection compared to uninfected controls (2,000 eggs per pig, 21 days post-infection) (Li et al., 2012). Our previous study also identified *Lachnospiraceae* as negatively associated with the three major STH infections, in human cohorts from both Liberia and Indonesia (Rosa et al., 2018). Another previous study of humans from India noted a significant negative association with *Lachnospiraceae* OTU and whipworm infection (Huwe et al., 2019). We confirmed a significant negative association with whipworm infection by RT-qPCR in mice (*P* = 0.005; **Supplementary Figure S6**). *Blautia* in the human intestinal microbiome has been shown to be associated with visceral fat accumulation in adults (Ozato et al., 2019) and with reduced death from Graft-versus Host Disease (GVHD) (Jenq et al., 2015), but its functional association with helminths is not well understood. The results presented here highlight the need for future research into its interactions with both helminths and the intestinal microbiome of the host.
(ii) *Clostridium sensu stricto* 1 (**Supplementary Figure S7**) was negatively associated with infection for at least one OTU in each comparison, however, among mice (but not humans), several OTUs and the genus as a whole showed a significant positive association with infection, both at baseline and following deworming, suggesting multiple roles for different species from this genus and an unclear relationship for the genus as a whole. Additionally, two OTUs from this genus showed lower abundance in the infected untreated mouse control cohort, further complicating its potential association with whipworm infection. In children from Ecuador, *Clostridium sensu stricto* was significantly less abundant in infected compared to uninfected individuals (Cooper et al., 2013).

**Figure 12:**
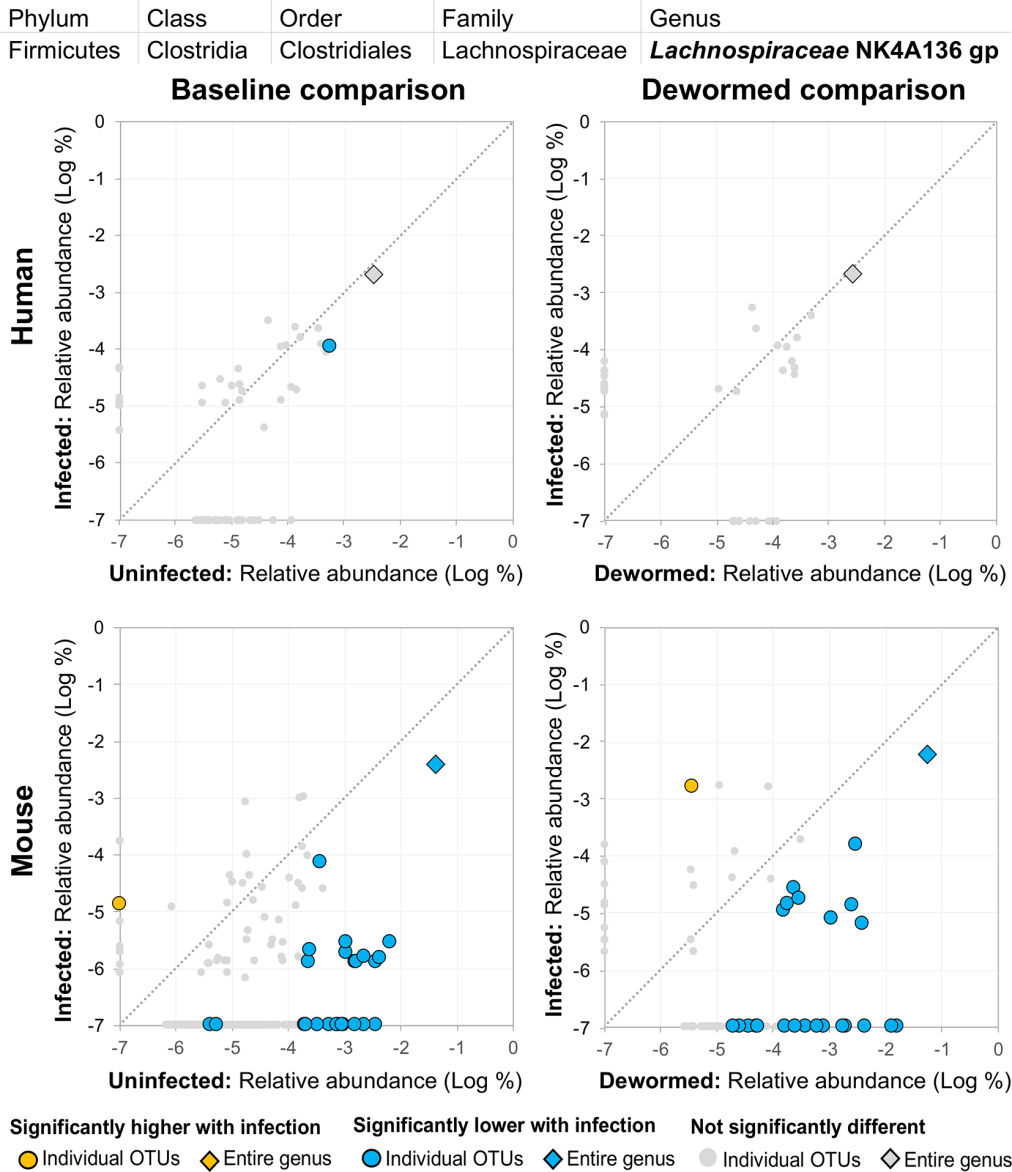
The relative abundance (%) of all *Lachnospiraceae* NK4A136 group (genus) OTUs in each differential comparison among samples from whipworm-infected and uninfected humans and mice. Significant differentially abundant OTUs in each comparison (as identified by LEfSe) are colored in orange (higher with infection) and blue (lower with infection). The sum of all OTUs in the genus is represented with a diamond symbol. This is one of three genera with at least one OTU significantly lower in infection in three of four comparisons.

Three bacterial genera were represented by OTUs that were significantly less abundant in the intestines of infected humans and mice compared to uninfected individuals, and significantly more abundant following deworming (negatively associated with infection) in mice but not significantly in humans:

(i) *Lachnospiraceae* NK4A136 group (**Figure 12**) is not described in association with intestinal parasitic infection in the literature, but is another member of the *Lachnospiraceae* family along with *Blautia* (described above). However, unlike for the other genera, there were many OTUs for *Lachnospiraceae* NK4A136 group which followed the same trend over the time period in the control mouse samples (the entire genus and five OTUs in uninfected untreated mice, and six OTUs in uninfected treated mice, although no differences in the human control samples or in the infected untreated mice). The identification of two different *Lachnospiraceae* members (*Blautia* and *Lachnospiraceae* NK4A136) among just five genera overall that consistently showed a negative association with infection across species highlights the importance of this bacterial family, which has also been observed to have a significant negative association with whipworm infection among humans from Malaysia (Lee et al., 2019) and mice with chronic whipworm infections (represented by the species *Roseburia* (20-egg infection, C57BL/6 mice, 35 days post-infection) (Holm et al., 2015)).
(ii) *Ruminococcaceae* UCG 014 (**Supplementary Figure S8**), like *Clostridium sensu stricto* 1 (above), was significantly represented by individual OTUs that were negatively associated with whipworm infection, but the entire genus is positively associated with infection in mice, highlighting a complicated relationship with infection. One OTU also increased over the deworming time period in uninfected treated mice. *Ruminococcaceae* showed a significant negative association (after 27 days) with chronic whipworm infection in a previous mouse study (Holm et al., 2015).
(iii) *Prevotella* 9 was previously described here as being positively associated with infection (**Figure 6**). However, although some OTUs from this genus were shown to have an overwhelmingly positive association with infection, at least one OTU in each of the three comparisons described for this genus were negatively associated with infection. This suggests the existence of different roles and functions for different species or strains within this genus.

A negative association with infection was consistently shown in both humans and mice for five taxa, four of which belong to the order *Clostridiales* (*Blautia*, *Clostridium sensu stricto* 1, *Lachnospiraceae* NK4A136 group, and *Ruminococcaceae* UCG 014), which had been described in association with infection in previous studies (as described above for individual genera). The *Clostridia* class that *Clostridiales* belongs to was the only taxa found to be negatively associated with whipworm/*Ascaris* infections in school children from Ecuador (Cooper et al., 2013). Following albendazole treatment of STH (hookworm and *Ascaris*)-infected patients in Kenya, there was a significant increase observed in *Clostridiales* (Easton et al., 2019), supporting the post-deworming increase of the order that we observed. Here, we highlighted specific genera, particularly those from the *Lachnospiraceae*, that were negatively associated with whipworm infection, both before infection and after infection, many of which are supported by previous additional human and mouse studies (as described above for individual genera).

## Conclusions

Interactions between the whipworm and the host intestinal microbiome where it lives are complicated and often identified inconsistently across the literature. Here, for the first time, we undertook a combined 16S rRNA gene OTU analysis to consistently identify bacterial taxa that were associated with infection of two closely related whipworm species in both human and mouse hosts. While many of the taxa identified by this analysis were consistent with the results of various human and/or mice studies in the literature, we validated these previous identifications by analyzing two host species in a combined bioinformatic analysis comparing different uninfected vs infected individuals at baseline as well as before and after deworming. Despite substantial differences in intestinal microbial ecology between these species, using this approach, we validated the use of murine whipworm infection as a model to evaluate whipworm-induced disruption in the human intestinal microbiome and subsequent restoration after deworming. While the specific mechanistic roles and host-parasite interaction consequences of identified microbiome genera are not necessarily conserved across the hosts, we identified 11 specific bacterial genera of interest for further association and mechanistic studies (particularly *Escherichia* and *Blautia*) which we suggest can be explored using a murine model of human whipworm infection. Overall, this novel approach to cross-species analysis provides a valuable tool to study the interaction between whipworm infection and the host intestinal microbiome.

## Data Availability Statement

The data presented in the study are deposited in the National Center for Biotechnology (NCBI) BioProject repository (https://www.ncbi.nlm.nih.gov/bioproject/), accession number PRJNA679627. **Supplementary Table S1** and **Supplementary Table S2** contain all relative abundance data per OTU per species, and all statistical comparison results.

## Ethics Statement

The studies involving human participants were reviewed and approved by Institutional Review Boards at Washington University School of Medicine in St. Louis, University of Liberia, Monrovia, and Ethical Committee for Research of the University of Indonesia, Jakarta. Written informed consent to participate in this study was provided by the participants’ legal guardian/next of kin. The animal study was reviewed and approved by USDA Beltsville Area Institutional Animal Care Committee.

## Author Contributions

MM, JU, and PF designed the study. BR and MM wrote the manuscript. JM processed and mapped 16S rRNA reads. BR and CS performed bioinformatic analysis. BR prepared figures and tables. JU performed mouse experiments and EB performed DNA extractions. TS and LG produced the human datasets. KF and JK performed RT-qPCR experimentation. All authors contributed to the article and approved the submitted version.

## Funding

This research was supported by the National Institute of Health: National Institute of Allergy and Infectious Diseases (NIAID) grant number AI081803-06A1.

## Conflict of Interest

The authors declare that the research was conducted in the absence of any commercial or financial relationships that could be construed as a potential conflict of interest.
