# Supplementary material for: Whipworm-Associated Intestinal Microbiome Members Consistent Across Both Human and Mouse Hosts
Source: Front Cell Infect Microbiol. 2021 Mar 11;11:637570. doi: 10.3389/fcimb.2021.637570 (PMC7991909; doi:10.3389/fcimb.2021.637570)
Supplement: Supplementary file 2 [file DataSheet_2.docx]

Supplementary Material

1. **Supplementary Figures**

**Supplementary Figure S1**. Sample cohorts used for differential intestinal microbiome analysis control comparisons. All control comparisons utilized the same individuals compared across the deworming time range.

**Supplementary Figure S2**. (A) MDS, NMDS and tSNE-based clustering of samples based on their normalized microbiome abundance profiles are shown for human and mouse. Red lines connect the same individual before and after deworming. (B) MDS clustering indicating human sample metadata (gender and age).

**Supplementary Figure S3**. MDS, NMDS and tSNE-based clustering of samples based on their normalized microbiome abundance profiles are shown for human and mouse, including control cohorts.

**Supplementary Figure S4**. (A) Relative abundance (% of total microbiome) of each bacterial class across all samples in humans and mice. Each column represents one sample in each sample group. OTUs which were unclassified at the class level are not shown.

**Supplementary Figure S5**. Taxonomic groups represented by at least a significant differentially abundant OTU in each of the four comparisons. Taxa counts were shown at the (**A**) OTU (**B**) species, (**C**) genera, (**D**) family, (**E**) order, (**F**) class and (**G**) phylum levels.

**Supplementary Figure S6**. RT-qPCR results for taxa of interest, tested in the mouse samples and in human samples from Indonesia that were infected with only *Trichuris* followed by deworming. N/S = Not significant according to a two-tailed T-test (unequal variance).

**Supplementary Figure S7**. The relative abundance (%) of all *Clostridium sensu stricto 1* (genus) OTUs in each differential comparison among samples from whipworm-infected and uninfected humans and mice. Significant differentially abundant OTUs in each comparison are colored in orange (higher with infection) and blue (lower with infection). The sum of all OTUs in the genus is represented with a diamond symbol. This is one of two genera with at least one OTU significantly lower in infection in all four comparisons.

**Supplementary Figure S8**. (**A**) The relative abundance (%) of all *Ruminococcaceae* UCG 014 (genus) OTUs in each differential comparison among samples from whipworm-infected and uninfected humans and mice. Significant differentially abundant OTUs in each comparison are colored in orange (higher with infection) and blue (lower with infection). The sum of all OTUs in the genus is represented with a diamond symbol. This is one of three genera with at least one OTU significantly lower in infection in three of four comparisons.

**2 Supplementary Tables**

**Supplementary Table S1** is provided in a separate MS Excel database, with A, B, C and D saved on separate tabs of the file:

**Supplementary Table S1A**: Sample metadata and groupings for each of the LEfSe comparisons.

**Supplementary Table S1B**: Complete OTU phylogeny and 16S rRNA read counts for each sample.

**Supplementary Table S1C**: Complete OTU phylogeny and normalized relative abundance (% of microbiome) for each sample.

**Supplementary Table S1D:** Complete LEfSe differential abundance statistics for every taxonomic level, for all sample comparisons.

**Supplementary Table S2**: qPCR primers used to amplify target genera of interest.

| Target Genus  (SILVA annotation) | Forward primer (5’-3’) | Reverse primer  (5’-3’) | Taqman 6FAM Probe (5’-3’) |
| --- | --- | --- | --- |
| *Prevotella* 2 | GCAACCGATGATGGCGAC | TCACGTTAGATGCCTGCTGTG | GAGTAACGCGTATCCAA |
| *Lachnospiraceae* NK4A136 group | ATACGTTTTAGTGGCGGACGG | TGAGTCATGCGACTCTGTGGTC | CGTGGGTAACCTGCC |
| *Blautia* | TCTGATGTGAAAGGCTGGGGCTTA | GGCTTAGCCACCCGACACCTA | N/A^*^ |
| *Collinsella* | AGGCVGGGGGTCRAAKSG | TTCGCCACCGGTGTTCC | AGGGTGGAACACCCGGT |
| Universal 16S rRNA (endogenous control) | TGGRACTGAGAYACGGYCC | TTACCGCGGCTGCTGG | CTACGGGAGGCAGCAG- |

**Blautia* was detected using the SYBR green assay
